# Assessment of Leptin Gene Polymorphism rs2060713 in Psoriasis Vulgaris

**DOI:** 10.1155/2014/845272

**Published:** 2014-01-28

**Authors:** Anthony Karpouzis, Gregory Tripsianis, Elisavet Gatzidou, Stavroula Veletza

**Affiliations:** ^1^University Clinic of Dermatology, School of Health Sciences, Democritus University of Thrace, 68100 Alexandroupolis, Greece; ^2^University Department of Medical Statistics, School of Health Sciences, Democritus University of Thrace, 68100 Alexandroupolis, Greece; ^3^University Laboratory of Medical Biology, School of Health Sciences, Democritus University of Thrace, 12 Chryssostomou Smyrnis Street, 68100 Alexandroupolis, Greece

## Abstract

Psoriasis is a lifelong disorder characterized by approximately 8-fold reduction of the duration of normal skin keratinocyte cell cycle and 2-fold increase of the number of dividing cells. Multiple genes, several environmental factors, and immune system alterations are involved in the pathogenesis of psoriasis. Hyperleptinemia is associated with psoriasis and leptin acts as an angiogenic factor. Angiogenetic processes precede the epidermal hyperplasia in psoriasis, indicating possible involvement of leptin in the pathogenesis of psoriasis. Leptin gene polymorphisms and their association with psoriasis have been given very little attention. We present a study of the rs2060713C/T genetic polymorphism in the pathogenesis of psoriasis vulgaris in 263 vulgaris patients and 252 unrelated matched healthy controls. No statistically significant differences were observed between patients and controls. A statistically nonsignificant trend was observed in males with the early onset type of psoriasis (11.1% C/T in patients versus 5.6% in controls) and in females with the late onset type of the disease (12.8% C/T in patients versus 3.3% in controls). Still, there is no hard evidence on correlation of psoriasis vulgaris with this polymorphism. Possible association with specific forms of the disease and either gender needs further investigation in larger studies.

## 1. Introduction

Psoriasis is a lifelong genetic disorder, whose appearance is influenced by both multiple genes and environmental factors. Genome-wide association studies have been carried out in order to establish causative genes of psoriasis vulgaris [1]. Genetic studies on psoriasis, performed with classical major histocompatibility complex alleles, indicated a strong association of early onset psoriasis with the HLA-Cw6, tightly linked to PSORS1. Genetic linkage analyses identified over 20 possible loci associated with psoriasis vulgaris. Association of the disease with genetic polymorphisms in specific candidate genes suggested their possible involvement in psoriasis vulgaris pathogenesis [1].

Leptin is a 16-kilodalton hormone, produced by adipocytes, in both subcutaneous and visceral fat, circulating in the serum, in either free or bound form. Leptin acts through the leptin receptor, a single-trans-membrane receptor detected in several tissues. The human leptin gene is situated on chromosome 7. By fluorescence in situ hybridization, this gene was mapped to 7q31.3 [2].

Skin leptin upregulates the hypoxia-inducible factor-1a that in turn influences several genes encoding key regulators of angiogenesis and wound healing. Leptin acts as an angiogenic factor, promoting wound repair acceleration and skin regeneration [3].

In the setting of the inflammatory processes of psoriasis, modifications in the levels of several adipokines have been reported [4]: some studies, show plasma levels of adiponectin in psoriatic patients as either lower [5–9] or higher [10], while others [11] have found no significant differences. Increased resistin plasma levels in psoriatic patients are normalized after treatment [12]. Visfatin levels are elevated in psoriatic samples of peripheral blood mononuclear cells (PBMC) skin [4] and serum [10]. Decreased levels of retinol-binding protein-4 may play a protective role in psoriasis by preventing the development of insulin resistance and diabetes [10]. Chemerin, an adipokine structurally similar to the precursors of cathelicidin, is higher in psoriatic patients [13]. Leptin plasma levels have been reported to be increased in psoriatic patients [5, 13–16], even though some studies did not detect significant differences [10, 11], whereas psoriatic patients with high PASI have even more elevated plasma leptin levels [14]. Considering all these adipokine level modifications in the setting of the inflammatory processes of psoriasis, we proceeded to study possible involvement of the gene encoding leptin in psoriasis, searching for a possible correlation between a gene polymorphism and the disease.

Surprisingly, leptin gene polymorphisms have not been studied extensively. Most such studies concern polymorphism involvement in body mass and diabetes. A rather popular polymorphism, G-2548A, located upstream from leptin gene coding region, has been studied for possible involvement in psoriasis. No statistically significant differences were observed between patients and healthy controls [17]. However, other studies have detected significant differences accompanied by similarly varying levels of leptin [18, 19], thus indicating involvement of at least one leptin gene polymorphism in psoriasis.

Here we attempt to study the role of another leptin gene polymorphism, rs2060713, that has been previously involved in blood pressure and carotid intima-medial thickness [20].

## 2. Materials and Methods

### 2.1. Patients' Characteristics

Two hundred and sixty consecutive psoriatic patients, examined at the University Clinic of Dermatology of the University General Hospital of Alexandroupolis: 159 males (60.45%) and 104 females (39.54%), were included in this study. Patients were randomly selected, regardless of body mass characteristics. Patients' age ranged from 15 to 80 years with a median age of 50.12 ± 16.50 years. All patients were clinically diagnosed and suffered from psoriasis vulgaris. Regarding age of onset, 173 patients were characterized as early onset, first diagnosed before the age of 40. Psoriasis patients with accompanying disease were excluded from the study in order to avoid possible interactions with potentially coexisting genetically determined disorders. Two hundred and fifty two healthy unrelated subjects, free from psoriasis, individually matched to patients by both gender and age, were recruited as controls (159 (63.1%) males and 93 (36.9%) females). There were no significant differences in gender (*P* = 0.538) and age (*P* = 0.632) between patients and controls.

### 2.2. Methods

Genomic DNA from whole blood was isolated by salting out. The rs2060713 leptin gene polymorphism was genotyped by PCR-RFLP. Primers F′: 5′-CCAGGCCTTGATTAAAGGAG-3′ and R′: 5′-CATTTAGGAGCTGCCATTTTC-3′ generated a 272 bp long amplicon after 40 cycles of PCR at 94°C 30′′, 57°C 30′′, 72°C 1′. The PCR product was subsequently digested with restriction enzyme BsiHKA-I (Alw21I) that recognizes GWGCW/C, for 16 hrs at 37°C. Presence of C at the polymorphic site was associated with recognition site for the restriction enzyme that generated two bands, 157 bp and 115 bp; presence of T resulted in a single uncut 272 bp band [20]. Restriction digests were analyzed in 2.5% agarose gel electrophoresis.

Statistical analysis of the data was performed using the statistical Package for the Social Sciences (SPSS), version 19.0 (SPSS, INC, Chicago, IL, USA). The chi-square test was used to assess differences of genotype and allele frequencies between patients suffering from psoriasis and matched controls. It was also used to compare the observed frequency of each genotype with that expected for a population in the Hardy-Weinberg equilibrium. Multivariate unconditional logistic regression analysis was used to estimate age-adjusted odd rations (OR) and 95% confidence intervals (CI) as the measure of association of the studied polymorphism with the development of psoriasis. All tests were two tailed and statistical significance was considered for values less than 0.05.

All work has been conducted in accordance with the Declaration of Helsinki.

## 3. Results

Rs2060713 is localized within intron 1 of the leptin gene on chromosome 7 [2]. The distribution of rs2060713 genotypes and alleles in patients suffering from psoriasis vulgaris and matched healthy controls is shown in Table 1(a). Comparison of the entire cohort of psoriatic patients with controls revealed no statistically significant difference, regarding distribution of the rs2060713 genotypes and alleles (*P* = 0.346, OR 1.35; 95% CI: 0.73–2.50 and *P* = 0.357; OR 1.33, 95% CI: 0.73–2.43) respectively. The analysis was subsequently stratified according to subject's gender (Table 1(a)) and disease's age of onset (Table 1(b)). More specifically, within male gender, both genotypes and alleles, in patients versus controls, did not reach statistical significance (*P* = 0.413, OR 1.40; 95% CI: 0.62–3.15 for genotypes and *P* = 0.423, OR 1.38; 95% CI: 0.63–3.06 for alleles). Similarly, within female gender, statistically significant differences were not reached between psoriasis patients and matched healthy controls (*P* = 0.639, OR 1.26; 95% CI: 0.48–3.27 for genotypes and *P* = 0.648, OR 1.26, and 95% CI: 0.49–3.16 for alleles). For the totality of the subjects included in this study, the genotype distribution in both groups (patients and controls) was in Hardy-Weinberg equilibrium (*x* ^2^ = 0.711, df = 1, and  *P* = 0.399 for patients; *x* ^2^ = 0.387, df = 1, and  *P* = 0.534 for controls).

Comparison of the cohort of early onset psoriasis patients versus matched controls (Table 1(b)) showed no statistically significant difference between the two groups, regarding both genotypes and alleles (*P* = 0.364, OR 1.41; 95% CI: 0.67–2.98 and *P* = 0.376, OR 1.39; 95% CI: 0.67–2.22, resp.). Within male gender early onset psoriasis vulgaris, a trend of higher frequency of C/T genotype and T allele was observed in patients (*P* = 0.140, OR 2.12; 95% CI: 0.77–6.89 for genotypes and *P* = 0.149, OR 2.06; 95% CI: 0.76–5.59 for alleles).

In late onset psoriasis vulgaris, no statistically significant difference was ascertained between patients and controls, regarding distribution of both genotypes and alleles (*P* = 0.72, OR 1.22; 95% CI: 40–3.68 and *P* = 0.730, OR 1.21, and 95% CI: 0.410–3.560, resp.). We also observed a weak trend of C/T genotype and T allele superiority in late onset female patients (*P* = 0.166, OR 4.27; 95% CI: 0.47–38.62 for genotypes and *P* = 0.176, OR 4.04; 95% CI: 0.46–35.5 for alleles).

## 4. Discussion

Genome-wide association studies (GWAs), used to identify genes possibly causative for psoriasis, may explain only a small proportion of psoriasis heritability and new gene variants may account for missing heritability. It is also possible that the input of certain leptin gene variants in the pathogenesis of psoriasis may be limited. However, to date, no objective and widely accepted criteria have been established to determine the degree of involvement of a gene in this disease [21].

In a previous study of rs2060713 [20] only 3 T/T homozygotes had been observed among 1373 studied individuals. Similarly, we encountered no T/T homozygotes in our present study and this was expected because of the low frequency of this allele (<5%).

As patients were selected randomly, regardless of body weight, we speculate that a different selection of psoriasis patients with similar body mass might have yielded different results, however, this experiment was designed differently. Leptin has been correlated with the histogenesis of psoriasis and involved in alterations of endothelial cell morphology and epithelial hyperplasia [3, 18, 22]. Estrogen regulates the action of leptin in endothelial cells [23]; it has been suggested that patients with psoriasis and hyperleptinemia tend to be of female gender and that those female patients manifested obesity and metabolic syndrome [16]. Moreover, 2-methoxyestradiol, an endogenous metabolite of estrogen, is an *in vitro* inhibitor of proliferation and migration of endothelial cells as well as of angiogenesis [24], all characteristic of psoriasis [25]. This involvement of sex hormones in psoriasis may provide ground for interpretation of gender differences in genotype distributions observed in psoriasis, where a trend of both C/T genotype and T allele frequency superiority was observed in male patients with early onset psoriasis and female patients with late onset psoriasis; however, no statistically significant differences were observed between any group or subgroup of patients and controls. It is possible that one or both of the observed trends may or may not be verified or become statistically significant in a larger study. However, these trends indicate some possible tendency regarding the eventual dependence either of the time of onset of the disease (severe early form or less severe late form) or of the male/female gender on more specific pathogenetic mechanisms for different clinical expressions of psoriasis.

## 5. Conclusion

Leptin gene polymorphism rs2060713 has not been statistically significantly associated with psoriasis vulgaris. An observed trend towards a possible association of this polymorphism with psoriasis' time of onset and/or patient's gender may suggest a possible contribution of the rare allele to particular clinical patterns of the disease.

## Figures and Tables

**Table 1a:** (a)

	Controls	Patients	*P* value	OR (95% CI)
Male gender				
Genotype			0.413	
C/C	148 (93.1)	144 (90.6)		ref.
C/T	11 (6.9)	15 (9.4)		1.40 (0.62–3.15)
T/T	0 (0.0)	0 (0.0)		—
Allele			0.423	
C	307 (96.5)	303 (95.3)		ref.
T	11 (3.5)	15 (4.7)		1.38 (0.63–3.06)
Female gender				
Genotype			0.639	
C/C	85 (91.4)	93 (89.4)		ref.
C/T	8 (8.6)	11 (10.6)		1.26 (0.48–3.27)
T/T	0 (0.0)	0 (0.0)		—
Allele			0.648	
C	178 (95.7)	197 (94.7)		ref.
T	8 (4.3)	11 (5.3)		1.24 (0.49–3.16)
Total				
Genotype			0.346	
C/C	233 (92.5)	237 (90.1)		ref.
C/T	19 (7.5)	26 (9.9)		1.35 (0.73–2.50)
T/T	0 (0.0)	0 (0.0)		—
Allele			0.357	
C	485 (96.2)	500 (95.1)		ref.
T	19 (3.8)	26 (4.9)		1.33 (0.73–2.43)

**Table 1b:** (b)

	Early onset				Late onset			
	Controls	Patients	*P* value	OR (95% CI)	Controls	Patients	*P* value	OR (95% CI)
Male gender								
Genotype			0.140				0.461	
C/C	102 (94.4)	96 (88.9)		ref.	46 (90.2)	48 (94.1)		ref.
C/T	6 (5.6)	12 (11.1)		2.12 (0.77–5.89)	5 (9.8)	3 (5.9)		0.58 (0.13–2.55)
T/T	0 (0.0)	0 (0.0)		—	0 (0.0)	0 (0.0)		—
Allele			0.149				0.471	
C	210 (97.2)	204 (94.4)		ref.	97 (95.1)	99 (97.1)		ref.
T	6 (2.8)	12 (5.6)		2.06 (0.76–5.59)	5 (4.9)	3 (2.9)		0.59 (0.14–2.53)
Female gender								
Genotype			0.725				0.166	
C/C	56 (88.9)	59 (90.8)		ref.	29 (96.7)	34 (87.2)		ref.
C/T	7 (11.1)	6 (9.2)		0.81 (0.26–2.57)	1 (3.3)	5 (12.8)		4.27 (0.47–38.62)
T/T	0 (0.0)	0 (0.0)		—	0 (0.0)	0 (0.0)		—
Allele			0.732				0.176	
C	119 (94.4)	124 (95.4)		ref.	59 (98.3)	73 (93.6)		ref.
T	7 (5.6)	6 (4.6)		0.82 (0.27–2.52)	1 (1.7)	5 (6.4)		4.04 (0.46–35.55)
Total								
Genotype			0.364				0.724	
C/C	158 (92.4)	155 (89.6)		ref.	75 (92.6)	82 (91.1)		ref.
C/T	13 (7.6)	18 (10.4)		1.41 (0.67–2.98)	6 (7.4)	8 (8.9)		1.22 (0.40–3.68)
T/T	0 (0.0)	0 (0.0)		—	0 (0.0)	0 (0.0)		—
Allele			0.376				0.730	
C	329 (96.2)	328 (94.8)		ref.	156 (96.3)	172 (95.6)		ref.
T	13 (3.8)	18 (5.2)		1.39 (0.67–2.22)	6 (3.7)	8 (4.4)		1.21 (0.41–3.56)
